# Interactions between MSCs and Immune Cells: Implications for Bone Healing

**DOI:** 10.1155/2015/752510

**Published:** 2015-04-27

**Authors:** Tracy K. Kovach, Abhijit S. Dighe, Peter I. Lobo, Quanjun Cui

**Affiliations:** ^1^School of Medicine, University of Virginia, Charlottesville, VA 22908, USA; ^2^Department of Orthopaedic Surgery, University of Virginia, Charlottesville, VA 22908, USA; ^3^Department of Medicine, University of Virginia, Charlottesville, VA 22908, USA

## Abstract

It is estimated that, of the 7.9 million fractures sustained in the United States each year, 5% to 20% result in delayed or impaired healing requiring therapeutic intervention. Following fracture injury, there is an initial inflammatory response that plays a crucial role in bone healing; however, prolonged inflammation is inhibitory for fracture repair. The precise spatial and temporal impact of immune cells and their cytokines on fracture healing remains obscure. Some cytokines are reported to be proosteogenic while others inhibit bone healing. Cell-based therapy utilizing mesenchymal stromal cells (MSCs) is an attractive option for augmenting the fracture repair process. Osteoprogenitor MSCs not only differentiate into bone, but they also exert modulatory effects on immune cells via a variety of mechanisms. In this paper, we review the current literature on both* in vitro* and* in vivo* studies on the role of the immune system in fracture repair, the use of MSCs in the enhancement of fracture healing, and interactions between MSCs and immune cells. Insight into this paradigm can provide valuable clues in identifying cellular and noncellular targets that can potentially be modulated to enhance both natural bone healing and bone repair augmented by the exogenous addition of MSCs.

## 1. Introduction

The normal process of fracture repair begins with an immediate inflammatory response as the innate immune system (macrophages, monocytes, neutrophils, and NK cells) responds with a variety of cytokines that recruit and activate several cell types, including osteoprogenitor mesenchymal stem cells (MSCs), to the site of injury [1, 2]. The adaptive immune response, primarily comprised of T and B lymphocytes, has important implications in the fracture healing process as well [3, 4]. For example, mice genetically deficient for adaptive immunity displayed accelerated bone healing. While some signals are mitogenic and proosteogenic, others function to inhibit osteogenesis and increase bone resorption, and it appears that a well-controlled, delicate balance of inflammatory factors is necessary for proper fracture repair [3–6]. Thus any process or systemic condition that alters this optimal inflammatory milieu, such as bone diseases like osteoporosis or severe trauma, steroid therapy, diabetes, or advanced age, can disrupt the normal fracture healing process, resulting in nonunions or delayed healing, pain, disfigurement, and loss of function. Approximately 5–15% of patients experience these complications and will require revision surgeries, prolonged hospitalization, and rehabilitation, all of which result in a high socioeconomic cost for society [7, 8].

**Figure 1:**
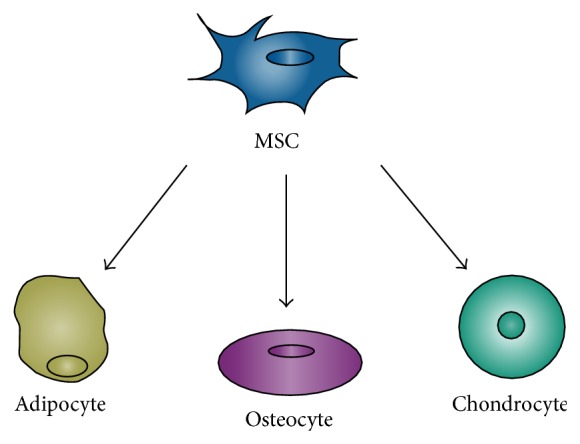
Multipotential differentiation of MSCs into adipogenic, osteogenic, and chondrogenic cell lineages. MSC = multipotent mesenchymal stromal cell.

Multipotent mesenchymal stromal cells (MSCs), also known as mesenchymal stem cells, have the capacity to differentiate into a variety of cell types (Figure 1), including adipocytes, chondrocytes, and osteocytes [9, 10]. Coupled with reports that allogeneic MSCs have immunoprivileged status and immunomodulatory properties, there has been considerable interest in exploring the use of these cells as a therapeutic option for bone repair. MSCs were initially isolated from bone marrow but are now known to exist in a wide range of tissues in the human adult, including brain, thymus, lung, liver, spleen, kidney, and dental pulp [11, 12]. MSCs have also been derived from embryonic tissues, such as Wharton's jelly and umbilical cord blood [13, 14]. Adipose-derived MSCs, in particular, pose an attractive option for cell-based therapy due to their relatively decreased morbidity during isolation and potential for expansion and differentiation [12].

MSCs are able to evade the host cell immune system due to their low expression of major histocompatibility complex (MHC) class I molecules and complete lack MHC class II molecules and other costimulatory molecules (CD40, CD40L, CD80, and CD86) required for immune cell stimulation [15–17]. Although the expression of MHC class I and II molecules can be upregulated by MSC exposure to inflammatory cytokines interferon-gamma (IFN-*γ*) and tumor necrosis factor-alpha (TNF-*α*), they are still unable to induce an immunological response [18]. There is also evidence that MSCs are able to modulate the immune system by a variety of mechanisms, including the release of soluble factors. Allogeneic MSCs have been shown to suppress T cell proliferation and antigen presenting cell maturation, as well as inducing a regulatory T cell phenotype that further suppresses the immune response* in vitro* [19–21]. Several* in vivo* studies using animal models, however, have yielded conflicting results as to whether allogeneic MSCs are immunoprivileged and maintain the ability to differentiate and proliferate [22–24].

Similarly immune cells recruited to injured bone can modulate osteogenic differentiation of osteoprogenitors. We have shown that Th1 immune response represented by enhanced expression of IFN-*γ* in the implants of allogeneic MSCs significantly inhibits expression of osteocalcin, Runx2, and alkaline phosphatase genes subsequently inhibiting bone formation [24]. Liu et al. have reported that combined action of IFN-*γ* and TNF-*α* that are primarily produced by activated T cells can induce apoptosis of MSCs [25]. These findings from animal studies were endorsed by a recent finding in human patients that CD8^+^ T cells in the circulation as well as in the fracture hematoma lead to delayed healing [26]. This continuous interaction between immune cells and MSCs during the bone repair process is one of the key factors that determine successful outcome of fracture healing. A new concept called “osteoimmunomodulation” is recently introduced which refers to alteration of immune response using various strategies to enhance bone repair [27]. It was reported that coating the magnesium scaffolds that are used very frequently for tissue engineering purposes, with *β*-tricalcium phosphate favored generation of M2 phenotype of macrophages which promoted osteogenic differentiation of MSCs [27]. M2 macrophages are known to suppress Th1 response and promote Th2 response. Another simple but very effective strategy was reported by Liu et al. [25]. Local delivery of aspirin inhibited IFN-*γ* and TNF-*α* activities and promoted bone regeneration [25]. These osteoimmunomodulatory strategies may become leading therapeutic interventions to enhance bone regeneration in near future.

In this review, we discuss the current understanding of the interactions between MSCs and the immune system in the context of osteogenesis and fracture repair.

## 2. Clinical Trials on Enhancement of Fracture Healing through Exogenous Addition of MSCs

Although there are numerous* in vitro* and* in vivo* studies published to date on the use of MSCs for regenerative medicine purposes, clinical trials using MSC-based approaches are limited due to medical and regulatory reasons [55]. As of September 2014, ten clinical trials were in process or completed investigating either autologous or allogeneic MSCs for fracture repair (http://www.clinicaltrials.gov) [55–60]. Most of the clinical trials used autologous MSCs that were culture expanded [56, 57] or bone marrow aspirate, concentrated using centrifugation [60, 61]. Since MSCs were delivered with the intention to increase the pool of osteoprogenitor cells and not as agents to modulate immune cells, potential change induced by MSCs in the local microenvironment of immune cells was not considered in relation to bone healing. It is also not clear whether allogeneic MSCs are as effective as autologous MSCs since no clinical trial has compared allogeneic and autologous MSCs. Therefore, existing data from the clinical trials throws very little light on the relationship between MSCs-induced immunomodulation and successful fracture healing.

## 3. *In Vivo* Animal Studies Demonstrating the Integral Role of Immune Cells in the Regulation of Natural Fracture Healing as well as the Success of Bone Repair through Transplantation of MSCs

### 3.1. Role of Immune Cells in Natural Fracture Healing

The fracture healing process begins with the formation of a soft callus that is subsequently mineralized and remodeled [3, 4, 62]. Successful fracture healing can be defined by adequate callus mineralization and the regeneration of biomechanical competence [3, 4, 62]. In the early phase of healing, the innate immune response plays a key role in the recruitment and activation of a variety of cell types that are critical in the fracture healing process, including MSCs [1, 2].

A study by Toben et al. investigated a standard closed femoral fracture in both wild-type (WT) and* recombination activating gene* knockout (*Rag1* ^−^ */* ^−^) mice that lack T and B cells [22]. It was found that fracture healing was significantly enhanced in* Rag1* ^−^ */* ^−^ mice suggesting detrimental functions of T and B lymphocytes on fracture healing. Higher numbers of lymphocytes were present during the repair process in the hematoma on day 3 and during formation of the hard callus on day 14 in the WT mice. Which of the two lymphocytes plays the predominant role in the regulation of bone repair remains a debate. It is reported that T cells are responsible for promoting bone resorption by induction of osteoclastogenesis via RANK-RANKL interactions with osteoclasts [63]. In* Rag1* ^−^ */* ^−^ mice, however, higher than normal numbers of osteoclasts were observed in the bone callus of these animals, even though they lacked the T lymphocytes to promote osteoclastogenesis [22]. The presumed reason for this increase in the number of osteoclasts is that they formed in response to the elevated osteoblast activity and bone formation in these animals [22].

Faster healing in these mice also correlated with lower levels of expression of TNF-*α*, a proinflammatory cytokine, in the callus [22]. This may favor bone formation since TNF-*α* can have proapoptotic effects on osteoblasts, and increased levels have been implicated in animal models of rheumatoid arthritis and other bone diseases characterized by excessive bony destruction [63].

After the initial innate inflammatory response, there appears to be a shift from proinflammatory to anti-inflammatory cytokines. The lymphopenic* Rag1* ^−^ */* ^−^ mice demonstrated an earlier and significantly higher expression of anti-inflammatory interleukin-10 (IL-10) [22]. The central role of IL-10 in promoting bone growth and accelerating fracture healing is further supported by studies showing that IL-10 regulates bone resorption, and its absence leads to osteopenia, mechanistic fragility, and malunion [64–66].

Another study carried out with *γ*/*δ* T cell deficient mice (*δ* T cell receptor- [TCR-] knockout mice) demonstrated superior quality of bony union with more osseous and chondral elements and mature bone marrow early on in the repair process compared to wild type controls [6]. TCR-knockout mice produced significantly lower levels of inflammatory cytokines IL-2, IFN-*γ*, and IL-6, at the fracture site [6]. Overall, the T cell deficient mice demonstrated improved biomechanical strength and stability compared to control animals, as evidenced by quantitative increases in osseous and chondral elements, increased gene expression of type II collaged, BSP, and BMP-2 [6].

A more recent study showed increased levels of terminally differentiated CD8^+^ effector memory T cells in the peripheral blood of humans with delayed fracture healing [26]. Furthermore, CD8^+^ T cells, as well as their cytokines IFN-*γ* and TNF-*α*, were enriched in the fracture hematoma of these patients [26]. In addition, CD8^+^ T cell-deficient mice demonstrated enhanced endogenous fracture healing, and a transfer of CD8^+^ T cells impaired the regenerative process [26]. This data supports the integral role that the adaptive immune response has on the outcome of endogenous bone regeneration [26].

In contradiction with the notion that the T cells inhibit bone healing, Nam et al. reported that T and B cell deficient* Rag1* ^−^ */* ^−^ mice displayed impaired fracture healing in comparison with wild type mice and the lack of T cells in the* Rag1* ^−^ */* ^−^ mice correlated with delayed osteoblast maturation and decreased bone formation [67]. Additionally, the proinflammatory cytokine IL-17, which is produced by Th17 lymphocytes (Th17 cells), was shown to be a key mediator in osteogenesis of the fracture healing process [67].

These studies suggest that T cells are inhibitory to fracture healing. The inflammatory cytokines produced by T cells, IFN-*γ* and TNF-*α*, play an important role in the T cells inhibition of bone regeneration. Further studies are required to elucidate roles of different subtypes of T cells as well as B cells in fracture repair.

### 3.2. Role of Immune Cells in Enhancement of Bone Formation through Exogenous Addition of Allogeneic and Syngeneic MSCs

#### 3.2.1. Use of Syngeneic MSCs

Liu et al. investigated the role of recipient T cells in MSCs-mediated osteogenesis in a calvarial defect in C57BL6 mice. This study demonstrated that proinflammatory T cells inhibited MSCs-induced bone formation via IFN-*γ* and TNF-*α* release [64]. IFN-*γ* induced downregulation of the runt-related transcription factor 2 (Runx2) pathway and enhanced TNF-*α* regulated MSC apoptosis (Figure 3) [64]. In addition, TNF-*α* was shown to convert IFN-*γ* activated nonapoptotic Fas to a caspase-8/3-associated apoptotic signal in MSCs via inhibition of NF-*κβ* signaling, leading to MSC apoptosis [64]. Furthermore, systemic infusion of T cells inhibitory Foxp3^+^ regulatory T cells (Tregs) significantly reduced levels of TNF-*α* and IFN-*γ* and resulted in improved MSCs-mediated bone regeneration and calvarial defect repair [64].

#### 3.2.2. Use of Allogeneic MSCs

Since MSCs isolated from senior people, diseased individuals, and females possess inferior osteogenic potential, it is advantageous to use allogeneic MSCs isolated from young, healthy males to enhance bone repair in these populations. However, some of the studies conducted in animal models suggest that use of allogeneic MSCs is not feasible owing to the immune response of the recipient host to transplanted MSCs.

An early study demonstrated a greater proportion of host-derived CD8^+^ T cells and NK cells infiltrate in the implants of MSCs implanted subcutaneously in allogeneic MHC-mismatched mice compared to syngeneic controls [68].

In another study by Nauta et al., bone marrow transplantation was performed with or without host or donor MSCs in allogeneic murine recipients [69]. The addition of host MSCs significantly increased the long-term engraftment associated with tolerance to host and donor antigens [69]. The infusion of donor MSCs, on the other hand, was associated with significantly increased rejection of allogeneic bone marrow cells and the induction of a memory T cell response [69]. This suggests that although autologous MSCs promote bone marrow engraftment* in vivo*, allogeneic MSCs are not intrinsically immunoprivileged [69].

In a murine model of allogeneic heart transplantation, MSCs from MHC mismatched allogeneic donors were implanted at various doses with and without cyclosporine A administration [70]. MHC mismatched MSCs not only failed to prolong allograft survival, but tended to accelerate allograft rejection [70]. Subsequently, MSC injections were ineffective at prolonging allograft survival and may even contribute to rejection [70]. Furthermore, in this study the immunosuppressive effect of cyclosporine A was abrogated by allogeneic MSCs, indicating a potential interaction* in vivo* between allogeneic MSC and cyclosporine A activities which is generally not observed* in vitro*.

The immunosuppressive potential of MSCs* in vivo* was tested by examining their ability to construct ectopic bone in both syngeneic and allogeneic murine recipients [71]. MSCs derived from bone marrow, placenta, and umbilical tissue were implanted with demineralized bone matrix under the kidney capsule. Bone formation was observed in only the syngeneic hosts, whereas the allogeneic hosts experienced transplant rejection. This data supports the argument for strong immunogenicity of MSCs in allogeneic recipients* in vivo* [71].

Our group has shown that cloned MSCs isolated from Balb/c mice could not induce ectopic bone formation in allogeneic B6 mice but bone formation was observed in syngeneic Balb/c mice and allogeneic mice lacking T and B cells. Expression of osteogenic genes (alkaline phosphatase, osteocalcin, and Runx2) was severely inhibited in allogeneic implants in comparison with syngeneic setting [24]. We also demonstrated a significant increase in numbers of T and B lymphocytes and macrophages recruited to the site of MSC implants in allogeneic hosts compared to the syngeneic group. Additionally, MSCs were shown to induce a larger proportion of Treg cells in the syngeneic group compared to the allogeneic group [24]. The Th1 immune response seems to be responsible for inhibiting osteogenesis in the allogeneic hosts, as evidenced by significantly increased levels of IFN-*γ*, the signature cytokine for the Th1 immune response [24].

In a more recent study of allogeneic versus autogeneic MSC implantation in rhesus macaques, increased production of NK, B and T cell subsets, and allo-specific antibodies was detected in the peripheral blood of those animals that received injection of the allogeneic MSCs targeted to caudate nucleus of the brain [72]. The magnitude and nature of the immune response correlated with the degree of MHC class I and II mismatch between the donor and recipients [72]. However, secondary antigen challenge did not elicit a measureable immune response in those recipients of allogeneic MSCs. Thus it was concluded that MSCs are weakly immunogenic in MHC mismatched individuals, which has implications for durable engraftment [72].

In a rat model of knee meniscus regeneration, the effects of autogeneic and allogeneic transplantation of synovial MSCs into rats with anterior meniscus defects were investigated [73]. The autogeneic group demonstrated a greater degree of meniscus regeneration than major mismatched transplant recipients at four weeks after transplant [73]. The number of macrophages and CD8^+^ T cells in the knee synovium was significantly lower in the autogeneic recipients compared to the allogeneic major mismatched group [73]. Results for the allogeneic minor mismatched recipients were comparable to the autogeneic group [73].

In complete disagreement with the studies mentioned above, several other investigations have obtained promising results on use of allogeneic MSCs. These animal studies suggest that allogeneic MSCs are immunoprivileged and it is possible to employ allogeneic MSCs for enhancement of bone repair.

In a study by Arinzeh et al., autogeneic and allogeneic MSCs were loaded onto a hollow cylinder of hydroxyapatite-tricalcium phosphate before being implanted into a critical-sized femoral defect of dogs. After radiographic, histological, and serum antibody evaluation at four, eight, and sixteen weeks, there were no adverse host responses detected at any time point [74]. Histological results between those defects filled with implants containing allogeneic MSCs and those filled with autologous MSCs were similar at 16 weeks, demonstrating callus formation across the length of the defect and lamellar bone in the pore of the implant at the host bone-implant interface [74]. Those implants filled with either autogeneic or allogeneic MSCs demonstrated a significantly greater amount of bone growth within the pore spaces than those implants that contained no MSCs [74].

In another study, autogeneic and allogeneic bone marrow MSCs were cultured in an osteogenesis-inducing medium and implanted into tibial shaft defects of mini-pigs [75]. There was no statistically significant difference in bone repair between the two groups [75]. There was a slight statistically significant increase in CD4 and CD8 T cells, as well as levels of IL-2 in both groups after transplantation, which likely indicates a traumatic inflammatory response [75]. This seemed to have no influence on the immunogenicity and osteogenic capacity of either autogeneic or allogeneic MSCs [75].

In a study of segmental radius defects in rabbits, bone marrow MSCs were culture expanded* in vitro*, and the defect was filled with hydroxyapatite alone, hydroxyapatite with autogeneic MSCs, or hydroxyapatite with allogeneic MSCs [76]. The groups with the addition of either autogeneic or allogeneic MSCs both demonstrated increased osteogenesis with superior quality cancellous bone and bone marrow formation as compared to the control group with hydroxyapatite alone [76]. No significant differences in results were observed between the autogeneic or allogeneic groups [76].

Similarly, allogeneic adipose-derived MSCs combined with demineralized bone matrix were shown to successfully regenerate ulnar bone defects in rabbits without generating an immunological response [77].

Yet another study yielded similar results using allogeneic peripheral blood derived MSCs or bone marrow derived MSCs combined with resorbable porous calcium phosphate substitute (Skelite) and implanted in bilateral critical-sized ulnar defect in rabbits [78]. Bone formation in the peripheral blood derived MSCs/Skelite group was comparable to the bone marrow derived MSCs/Skelite group, and both groups showed significantly enhanced bone regeneration when compared to controls [78].

A study of human adipose-derived MSCs embedded in fibrin glue and then implanted in a critical-sized defect in immunocompetent rat mandibles demonstrated a significantly higher amount of ossification on radiographic examination when compared to controls [79]. The level of bone regeneration using the adipose-derived MSCs was shown to be comparable to the gold standard of autologous bone grafting [79]. Similarly, another study using human MSCs in a hydroxyapatite-tricalcium phosphate scaffold implanted into a critical-sized calvarial defect in nude mice resulted in enhanced osteogenesis when compared to controls with scaffold alone [80].

Another study evaluated ectopic bone formation induced by allogeneic MSCs that were seeded onto a *β*-tricalcium phosphate scaffold and implanted subcutaneously into dogs [30]. There was no significant difference found in the number of CD4 T cells, CD8, T cells, and CD4/CD8 T cell ratios between the recipients of allogeneic MSCs and those that received either scaffold alone or scaffold seeded with autogeneic MSCs [30]. Both the autogeneic and allogeneic implants yielded subcutaneous ectopic bone formation, unlike the control group with scaffold alone [30].

A study by Lee et al. tested the immunogenicity of allogeneic human umbilical cord blood-derived MSCs via repeated intravenous injection into a humanized immunocompromised mouse model [81]. The human MSCs did not elicit an immunological response in the form of T cell proliferation or increased IFN-*γ* and TNF-*α* levels [81]. Additionally, mice that received intravenous injections of human peripheral blood mononuclear cells demonstrated lymphocyte infiltration in the lung and small intestine and reduced survival rates, while those that received MSCs demonstrated no such adverse events, suggesting the low immunogenicity of MSCs* in vivo* [81].

Similar to their effect on natural fracture healing, T cells, IFN-*γ*, and TNF-*α* inhibit bone formation induced by exogenously added MSCs. Treg cells that inhibit activities of T cells promote MSC-mediated bone formation. While more scientific studies addressing the controversial immunoprivileged status of MSCs are certainly needed, it is possible to draw a few inferences from existing published literature that can help to decide future direction of the research in this field. Intriguingly, out of seven studies [24, 68–73] that attest to the nonimmunoprivileged status of MSCs, six were performed in mice while only one study used a rhesus macaques model. On the other hand, out of nine studies [23, 30, 74–80] that demonstrated successful use of allogeneic (or xenogeneic) MSCs, six utilized large animal models (rabbits, pigs, and dogs) and one study used xenogeneic rats while two studies used immunocompromised xenogeneic mice. This observation suggests that mice are not good hosts to accept allogeneic MSCs in comparison with other animal models, but the mechanisms remain unknown at this time. Another interesting difference between two groups of studies was that all the studies that demonstrated successful use of allogeneic MSCs used a fracture model or bone defect model while all the studies demonstrating failure of allogeneic MSCs transplanted MSCs in tissues other than bone. It is necessary to investigate what factors in the inflammatory microenvironment at injured bone promote survival and differentiation of allogeneic MSCs.

## 4. *In Vitro* Studies on MSC Regulation of T Cells

It is widely believed that, upon transplantation, MSCs can evade the immune system of major histocompatibility complex- (MHC-) mismatched host since MSCs display low expression of MHC class I molecules and completely lack MHC class II molecules as well as other costimulatory molecules (CD40, CD40L, CD80, and CD86) required for immune cell stimulation. Although the expression of MHC class I and II molecules can be upregulated by MSC exposure to inflammatory cytokines interferon-gamma (IFN-*γ*) and tumor necrosis factor-alpha (TNF-*α*), they are still unable to induce an immunological response [18].

MSCs possess significant and diverse immunomodulatory properties that affect both the innate and adaptive immune systems. With regard to the adaptive immune system, MSCs have been shown to have direct immunosuppressive properties by inhibiting the activation and proliferation of effector T cells (both CD4^+^ and CD8^+^) via cell-to-cell contact and the elaboration of various soluble factors [18]. MSCs can also induce generation and the proliferation of T-cell inhibitory regulatory T (Treg) cells [18]. Both the direct suppression of MSCs on effector T lymphocytes and the indirect suppression mediated by MSC induction of Treg proliferation have been well documented in* in vitro* studies, which will be reviewed later in this section. Of note, MSCs seem to require “licensing” or activation by exposure to inflammatory cytokines such as IFN-*γ*, TNF-*α*, and interleukin- (IL-) 1*β* prior to exerting their immunomodulatory effects [82–84].

Intriguingly, the abundance of mediators and proposed mechanisms suggests a complex interplay in which MSCs may be either immunosuppressive or immunogenic [82, 83]. The dominant effect seems to depend on the microenvironment of the cellular milieu as well as the ratio of MSCs to T lymphocytes. A high MSC to lymphocyte ratio is associated with an inhibitory effect on the immune response, whereas a low MSC to lymphocyte ratio is characterized by an enhanced proliferation of lymphocytes [85]. The immunomodulatory effects of MSCs on these T cell subsets also appear to occur in a dose-dependent fashion [85]. More recently, a new paradigm has been proposed in which MSCs can be polarized into two phenotypes based on the stimulation of specific toll-like receptors. TLR4 stimulation polarizes them into a proinflammatory phenotype whereas TLR3 stimulation of MSCs leads to immunosuppressive signature. The first proinflammatory and immunocompetent phenotype is denoted as MSC1, while MSC2 is used to denote MSCs possessing anti-inflammatory and immunosuppressive characteristics [29, 86].

### 4.1. T Cell Differentiation and Function

T helper (Th) cells are cytokine-producing CD4^+^ T cells that may differentiate into either of the well-defined subsets Th1 and Th2, depending on the peptides presented to them by major histocompatibility complex (MHC) class II molecules on antigen presenting cells (APCs) [87]. The differentiation of Th1 cells is guided by interleukin- (IL-) 2, IL-12, and interferon-gamma (IFN-*γ*). The main effector cytokines of Th1 cells are IFN-*γ* and tumor necrosis factor-beta (TNF-*β*). Th1 cells function to recruit macrophages, as well as induce the production of immunoglobulin (Ig) G by B cells. Th2 cell differentiation is guided by IL-4, and their main effector cytokines are IL-4, IL-5, and IL-13. The primary effector function of Th2 cells is to recruit eosinophils, basophils, and mast cells [87]. Th2 cells also mediate B cell antibody class switching to IgE and IgG. Cytotoxic T lymphocytes (CTLs) are CD8^+^ T cells whose differentiation is guided by the presentation of an antigen by an MHC class I molecule upon an antigen presenting cell (APC), as well as costimulation by either CD80 or CD86 on the same APC. Once activated, IL-2 stimulates CTL proliferation. Th17 cells are a developmentally distinct type of T helper cell whose differentiation is guided by TGF-*β*, IL-6, and IL-21. The main effector cytokine of Th17 cells is IL-17, which plays an antimicrobial role at epithelial and mucosal barriers. Regulatory T cells (Tregs) are a subset of CD4^+^ T lymphocytes that are characterized by the expression of cell surface receptor CD25, as well as the presence of high levels of transcription factor forkhead box P3 (Foxp3). Tregs function to modulate the immune system and maintain tolerance to self-antigens. The mechanism by which Tregs carry out their regulatory function is not well understood, though immunosuppressive cytokines TGF-*β* and IL-10 are well implicated as role players [87].

### 4.2. MSCs Inhibit T Lymphocyte Proliferation

Both murine and human MSCs have been shown to inhibit the proliferation of stimulated T lymphocytes* in vitro* in both allogeneic and autologous settings [86]. The immunosuppressive effect of MSCs on allogeneic and autologous T lymphocyte proliferation is dependent on a high MSC to lymphocyte ratio and soluble factors [86]. Schurgers et al. demonstrated similar dose-dependent immunosuppressive effects of MSCs on anti-CD3-induced allogeneic T cell proliferation. However, MSCs did not show immunosuppressive effects* in vivo*. The authors demonstrated a role for inducible nitric oxide (iNOS), programmed death ligand-1 (PD-L1), and prostaglandin E2 (PGE2), but not indoleamine 2,3-dioxygenase (IDO), in T cell inhibition* in vitro* [88].

**Figure 2:**
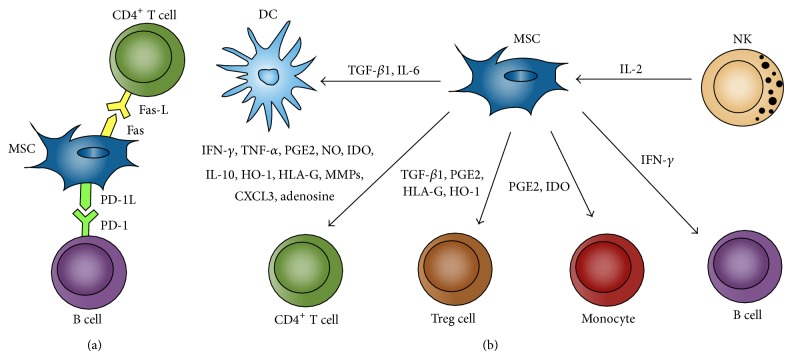
Mechanism of MSC modulation of immune cells. (a) Direct cell-cell contact, (b) soluble factors interactions.

**Figure 3:**
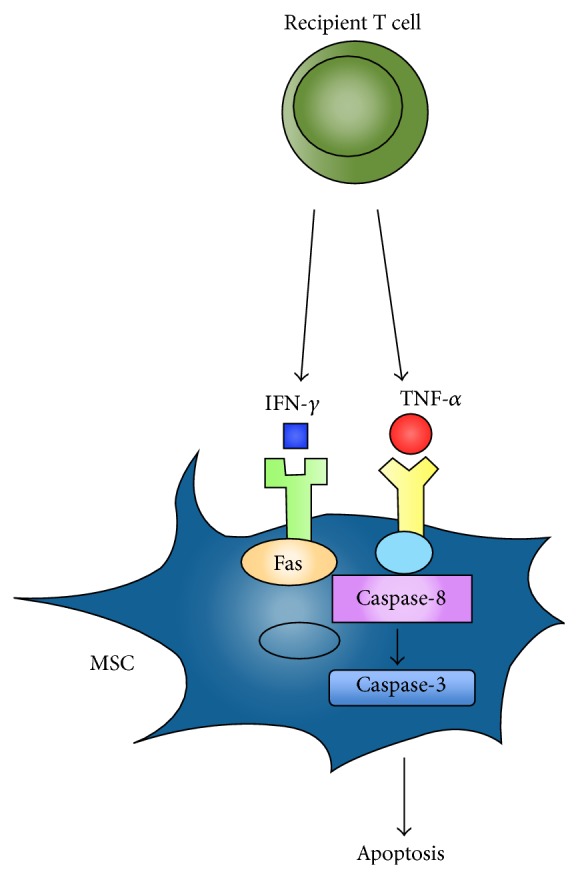
Immune cell modulation of MSCs. Combined action of IFN-*γ* and TNF-*α* induces apoptosis of MSC.

There are a variety of proposed mechanisms by which MSCs mediate this T cells inhibition (Table 1, Figure 2). Initial data demonstrated that MSCs do not induce T-cell apoptosis but instead inhibit proliferation by inducing T cell cycle arrest in the G0 phase [36, 89, 90]. However, a recent study demonstrated that MSCs also could induce transient T cell apoptosis mediated by the FAS ligand- (FASL-) dependent FAS pathway [34]. Additionally, MSC immunosuppression seems to be mediated in part by the activation of nuclear factor kappa B (NF-*κ*B) signaling in MSCs, and this pathway is activated by TNF-*α* generated by the TCR stimulation of allogeneic T cells [35, 91]. MSCs have been shown to inhibit the effects of CTLs by limiting their proliferation rather than their cytolytic activity. The mechanism by which MSCs exert this immunosuppressive effect on CTLs involves B7-H4, a negative costimulatory molecule that induces cell cycle arrest and inhibits the nuclear translocation of nuclear factor kappa beta (NF-*κβ*) [92, 93].

Human bone marrow MSCs have also been shown to inhibit antigen-dependent CD4^+^ and CD8^+^ T cell proliferation in an allogeneic setting* in vitro* [94]. The suppressive effect of MSCs on CD4^+^ and CD8^+^ T cells is due to inhibition of T cell proliferation, as opposed to effector function, as the cytotoxicity of T cells seems to be unaffected [95]. Human MSCs were shown to downregulate level of CD8 expression significantly on allogeneic CD8^+^ T cells. The mechanism involved induction of a tolerogenic monocyte phenotype (lower expression of costimulatory molecules CD80 and CD86, higher expression of inhibitory receptors ILT-3 and ILT-4) representing an alternative mechanism for immunosuppression [96]. A more recent study confirmed that allogeneic MSCs inhibit the proliferation of CD8^+^ T cells in a mixed lymphocyte reaction [31].

Conflicting data exists as to whether MSCs are susceptible to lysis by activated CTLs. MSCs were shown to be resistant to lysis by allogeneic effector CTLs, and this was associated with inefficient upregulation of CD25 surface molecules on activated cells, as well as a lack of IFN-*γ* and TNF-*α* production by the CTLs [21]. Allogeneic MSCs were shown to be susceptible to lysis by CD8^+^ CTLs, whereas autologous MSCs were resistant to CD8^+^ CTL lysis [97]. Another study demonstrated that CD8^+^ T cells were capable of HLA specific lysis of allogeneic BMSCs, and that this effect was augmented by exposure to IFN-*γ* [37].

### 4.3. MSCs Induce Treg Proliferation

As a part of exerting their immunosuppressive effects, MSCs are able to induce the generation of classic CD4^+^CD25^+^Foxp3^+^ Tregs. Numerous mediators and mechanisms have been proposed to be involved in MSC promotion of this classic Treg phenotype. Allogeneic MSCs have been shown to induce Foxp3 and CD25 expression in CD4^+^ T cells through direct cell contact followed by production of MSC-derived TGF-*β*1 and PGE2 [38, 82]. Another study in which MSCs were shown to promote the generation of CD4^+^CD25^+^Foxp3^+^ Tregs also supports the role of TGF-*β*1 in the mechanism of induction [46]. Selmani et al. demonstrated that human leukocyte antigen-G5 (HLA-G5) was required for MSC promotion of Tregs in an allogeneic setting [98]. Notch1 signaling has been implicated in the mechanism of MSC induction of Treg differentiation from allogeneic, activated CD4^(+)^ T lymphocytes given that MSCs express the Notch1 ligands Jagged1, Jagged2, and Delta-Like (DLL) 1, 3, and 4 [39]. Luz-Crawford et al. demonstrated that MSCs were able to suppress the proliferation, activation, and differentiation of allogeneic Th1 and Th17 cells, and this immunosuppressive effect was associated with the induction of CD4^+^CD25^+^Foxp3^+^ Treg cells [42]. Additionally, when MSCs were cocultured with allogeneic Tregs, MSCs seemed to augment the immunosuppressive capability of the Treg cells, and this effect was accompanied by an upregulation of the PD-1 receptor on Tregs via the production of IL-10 [43]. Yet another study demonstrated that MSC production of heme oxygenase-1 (HO-1) is involved in Treg induction [99].

In addition, MSCs have been shown to induce epigenetic changes at the promoter of the FOXP3 gene locus in allogeneic Th17 cells that led the acquisition by Th17 cells to inhibit the proliferative response of activated CD4^+^ T cells* in vitro* [45]. In this same study, MSCs seemed able to promote the differentiation of proinflammatory Th17 cells into functional Tregs via chemokine receptor 6 (CCR6) [45]. Another study suggests that adenosine produced by MSCs could play a role in promoting the differentiation of Th17 cells into Tregs via the upregulation of CD39 [32].

### 4.4. Direct Cell-to-Cell Contact

Direct modulatory effects of MSCs on both autologous and allogeneic T lymphocytes via cell-to-cell contact have been well described* in vitro*, supported by the demonstration that MSCs express various integrins, intracellular adhesion molecules, and vascular cell adhesion proteins on their cells surface [16, 48]. Ren et al. provided further evidence for the necessity of cell-to-cell contact for the immunosuppressive effects of MSCs on T lymphocytes [49]. The expression of cell-to-cell adhesion molecules ICAM-1 and VCAM-1 by MSCs was positively correlated with the immunosuppressive effects of MSCs towards various subtypes of T cells [49]. Furthermore, the genetic deletion or functional blocking of these adhesion molecules led to significant reversal of MSC immunosuppressive effects [49].

One study proposes that MSC inhibition of allogeneic Th17 cell differentiation is mediated by PGE2 via the EP4 receptor and is dependent on cell-cell contact [50]. Another study supports the notion that cell-to-cell contact is necessary for the inhibition of Th17 differentiation, and that this is mediated specifically by the upregulation of programmed death-1 (PD-1) ligand expression on IFN-*γ* primed allogeneic MSCs [42].

Galectins are a family of cell surface proteins with a broad variety of functions, including the ability to bind neuropilin-1 (NP-1) on the surface of T cells and induce cell cycle arrest [51]. Allogeneic MSCs have been shown to constitutively express galectins, and these molecules help to mediate the immunosuppressive effect of MSCs [52]. Specifically, galectin-1 and galectin-3 were shown to inhibit T-cell proliferation, and genetic knockdown of these molecules resulted in a significant loss of immunomodulatory properties, specifically upon CD4^+^ and CD8^+^ T cell proliferation [51–53]. The effect of allogeneic MSCs on NK cells appeared to be unaffected by galectin-1 knockdown, however [53]. It was also found that the production of galectin-9 in allogeneic MSCs was strongly upregulated in the presence of proinflammatory cytokines IFN-*γ* and TNF-*α*, and this was associated with the antiproliferative effects that MSCs have on T cells [53].

### 4.5. Mechanism of MSCs Inhibition of T Cells through Soluble Mediators

Although it has been shown that cell-to-cell contact is necessary for MSC mediated immunosuppression, there are several experiments that have been performed demonstrating that both autologous and allogeneic MSCs also exert their immunomodulatory effects through the elaboration of soluble factors [48]. Given the plethora of mediators proposed, it is likely a combination of complex interplay between these factors and the specific inflammatory milieu that contributes to metabolic manipulation of the microenvironment and the overall immunomodulatory effects of MSCs [48].

It is important to recognize that there are some well-delineated differences between MSCs from different species. Notably, MSCs derived from mice produce nitric oxide (NO) via inducible nitric oxide synthase (iNOS) to suppress T cell proliferation [49]. NO has been shown to suppress the phosphorylation of signal transducer and activator of transcription-5 (STAT-5), which is a critical transcription factor for T cell activation and proliferation [49]. In contrast, human allogeneic MSCs exert this effect by the upregulation of indoleamine 2,3-dioxygenase (IDO), an enzyme involved in the catabolism of essential amino acid tryptophan to N-formylkynurenine [100]. Delarosa et al. demonstrated that human adipocyte-derived MSCs are activated by IFN-*γ* to express functional IDO in allogeneic settings [101]. Furthermore, IDO expression upon IFN-*γ* activation is essential for the immunosuppressive activity of allogeneic AMSCs, since IDO exerts its effects through the accumulation of tryptophan metabolites in the local microenvironment [101, 102].

#### 4.5.1. IFN-*γ*

Interferon-gamma (IFN-*γ*) is an inflammatory cytokine that plays a critical role in licensing allogeneic MSCs to inhibit activated T cell proliferation, and this process is IDO-dependent [103]. IFN-*γ* activates the synthesis of IDO and upregulates the expression of hepatocyte growth factor (HGF) and TGF-*β* by allogeneic MSCs [104]. When compared to unprimed MSCs, MSCs pretreated with IFN-*γ* and TNF-*α* were more effective at inhibiting T cell proliferation [47]. IFN-*γ* also plays a role in allogeneic MSC suppression of T lymphocyte effector functions, namely through the inhibition of Th1 cytokines (IFN-*γ*, TNF-*α*, and IL-2), and this process is mediated by the PD-1 ligand on MSCs [105].

#### 4.5.2. TNF-*α*

Tumor necrosis factor-alpha (TNF-*α*) is another inflammatory cytokine that has been shown to augment the immunomodulatory properties of MSCs. Studies have shown that TNF-*α*, along with IFN-*γ*, promotes the expression of HGF, PGE2, and COX-2 levels by allogeneic MSCs, contributing to the inhibition of proliferating T lymphocytes [106]. More recently, it has been demonstrated that TNF-*α* released by activated T cells binds to TNF-R1 on allogeneic MSCs, activating the NF-*κ*B pathway and contributing the immunosuppressive properties of MSCs [35, 91].

#### 4.5.3. IL-10

IL-10 is an anti-inflammatory cytokine produced by monocytes, Th2 cells, and Tregs. It functions to downregulate the expression of Th1 cytokines, MHC class II antigens, and macrophage costimulatory molecules. Allogeneic MSCs cocultured with either naïve or activated T cells have been shown to produce a significant amount of IL-10, and this was associated with significant suppression of T cell proliferation [100]. The addition of anti-IL-10 and anti-IL-10-receptor antibodies restored T cell proliferative capacity, providing further evidence for the critical role of IL-10 in allogeneic MSC immunosuppression of T lymphocyte proliferation [100]. Qu et al. demonstrated that allogeneic MSCs were able to inhibit Th17 differentiation* in vitro* via the secretion of IL-10 [107]. More recently, it has been shown that allogeneic MSCs cocultured with CD4^+^ T cells led to increased secretion of IL-10 by T helper cells [108]. Allogeneic MSCs are able to inhibit Th17 cell differentiation [107]. Since Th17 differentiation was restored when IL-10 was specifically neutralized or the expression of IL-10 by MSCs was downregulated by RNA interference, it has been suggested that this effect is mediated by IL-10 secretion by MSCs [107].

#### 4.5.4. PGE2

Prostaglandin E2 (PGE2) is an enzyme responsible for the metabolism of arachidonic acid and prostaglandin production [109]. PGE2 prevents the proliferation of T cells and inhibits production of cytokines such as TNF-*α* and IL-12 [33, 110]. It also downregulates MHC class II molecules on macrophage surfaces and skews the T helper differentiation towards a Th2 response with IL-4 and IL-5 production [28, 111]. High levels of PGE2 produced by allogeneic MSCs have been shown to inhibit the maturation of dendritic cells, as well as the proliferation of activated T cells and their subsequent proinflammatory cytokine production [40].

#### 4.5.5. HO-1

Heme oxygenase-1 (HO-1) is an inducible enzyme that catalyzes the first and rate-limiting step in the degradation of heme into biliverdin, iron, and carbon monoxide [41]. The products of heme metabolism produced by HO-1 during inflammation are associated with antiapoptotic, antioxidative, and anti-inflammatory effects [41]. HO-1 has been implicated as having a role in the mechanism of allogeneic MSC induction of Treg proliferation and IL-10 production [99]. Once MSCs have been licensed by inflammatory factors in a mixed lymphocyte reaction, however, there was substantial downregulation of HO-1, yet Treg induction as well as IL-10 production by MSCs was not affected [99]. This suggests that HO-1 plays an initial role in MSC immunosuppressive effects* in vitro*, but this is taken over by other molecules after alloreactive priming [99].

#### 4.5.6. Nitric Oxide

As mentioned earlier, NO has been shown to suppress the phosphorylation of signal transducer and activator of transcription-5 (STAT-5), which is a critical transcription factor for T cell activation and proliferation [49]. Another study demonstrated that NO production by allogeneic MSCs suppressed the proliferation of T lymphocytes via the inhibition of STAT5 phosphorylation, and that inhibitors of inducible NO synthase (iNOS) restored the proliferation of T cells [112]. The presence of cytokines TNF-*α* and IL-1*β* was shown to provoke the expression of high levels of iNOS by MSCs [49].

#### 4.5.7. HLA-G

MSCs have been shown to mediate their immunomodulatory effects via the production of soluble factor human leukocyte antigen-G (HLA-G) [54]. HLA-G secretion by allogeneic MSCs has been shown to suppress T cell proliferation in mixed lymphocyte reactions [54, 113]. Exogenous IL-10 was shown to stimulate HLA-G secretion and was shown to play a key role in allogeneic MSC inhibition of peripheral blood mononuclear cell response to phytohemagglutinin [44]. Another study demonstrated that HLA-G secretion by human allogeneic MSCs not only suppressed allogeneic T lymphocytes, but also induced the proliferation of CD4^+^CD25^+^Foxp3^+^ Tregs [98]. This same study also demonstrated that MSCs inhibit cell-mediated lysis and IFN*-γ* secretion by allogeneic NK cells [98].

#### 4.5.8. MMPs

Matrix metalloproteinases (MMPs) derived from allogeneic MSCs, in particular MMP-2 and MMP-9, have been shown to cause the cleavage of IL-2 receptor *α* (CD25) from the surface of activated T cells and thus the suppression of IL-2 production and T cell proliferation [62, 114].

#### 4.5.9. Chemokines

Chemokines CXCL1, 2, and 3 were shown to be induced in T cells cocultured with allogeneic MSCs. CXCL3, in particular, was associated with the inhibition of T cell proliferation and increased apoptosis [115].

#### 4.5.10. Adenosine

Both human and murine allogeneic MSCs have been shown to generate adenosine, which inhibits the proliferation of T lymphocytes by acting through its receptor A(2a) (ADORA2A) [116, 117]. MSCs upregulate CD39 and increase adenosine production to suppress activated T-lymphocytes [116].

## 5. *In Vitro* Studies on MSC Regulation of Other Immune Cells

### 5.1. Macrophages

Macrophages differentiate from monocytes into one of two main phenotypes—immunogenic M1 macrophages and immunosuppressive M2 macrophages. Monocytes are stimulated towards the M1 phenotype by bacterial products, such as lipopolysaccharide (LPS), and inflammatory cytokines [118]. These M1 macrophages function in the phagocytosis of cellular debris and pathogens and secrete IFN-*γ*, TNF-*α*, and IL-6, among other proinflammatory cytokines [118]. The M2 phenotype is induced by IL-4 and IL-13, secretes primarily IL-10, and functions in tissue repair [118]. Autologous and allogeneic MSCs have been shown to significantly suppress the production of inflammatory cytokines TNF-*α*, IL-6, IL-12p70, and IFN-*γ* by macrophages, while increasing the production of anti-inflammatory IL-10 and IL-12p40 [119–121]. This process seemed to be mediated by PGE2 [119–121]. Additionally, both autologous and allogeneic MSCs seemed to inhibit the upregulation of CD86 and MHC class II expression in LPS-stimulated macrophages, impairing their immunogenic effects on CD4^+^ T cell [103, 119]. More recent studies provide evidence that allogeneic MSCs promote the shifting of monocytes toward an anti-inflammatory M2 phenotype [122–124]. This M2 polarization induced by allogeneic MSCs may occur through the NF-*κ*B and STAT-3 pathways and involve IDO activity [103, 125]. Melief et al. suggest that the pathway involved in the shifting of monocytes towards the M2 phenotype is a necessary part of the ability of MSCs to induce Treg proliferation [122].

### 5.2. Dendritic Cells

Dendritic cells (DCs) are antigen presenting cells (APCs) that phagocytose and process antigens into peptides and present them via MHC molecules on their cell surface to prime T lymphocytes as part of the adaptive immune response [126]. DCs differentiate from monocytes and secrete IL-12, which aids in the differentiation of Th1 cells from naive CD4^+^ T cells. Allogeneic MSCs have been shown to impair the maturation of DCs from monocytes or CD34^+^ hematopoietic precursors, as well as their ability to secrete proinflammatory cytokines [127]. Additionally, allogeneic MSCs were shown to increase the release of anti-inflammatory IL-10, as well as inhibit the polarization of naïve CD4^+^ lymphocytes into Th1 cells [128, 129]. Similar to the mechanisms of immunosuppression on T lymphocytes, allogeneic MSC mediated inhibition of DC function appears to be dependent on cell-to-cell contact [130]. One study suggests that TGF-*β*1 production and the downregulation of DC costimulatory molecules (such as CD80, CD86, and CD40) are responsible for the inhibitory effect of MSCs on DCs [131, 132]. DCs that have been cocultured with either autologous or allogeneic MSCs also display the ability to induce classic Treg differentiation from naïve T cells [131, 132]. One study suggests that allogeneic MSCs cocultured with monocyte-derived DCs secrete growth-regulated oncogene chemokines that drive the DCs towards a myeloid-derived suppressor cell- (MDSC-) like phenotype [133]. More recently, it has been suggested that MSCs mediate the upregulation of the gene SOCS1 via IL-6, which instructs DCs to acquire a tolerogenic phenotype with a significant increase in the production of IL-10 and the ability to induce Treg and Th2 differentiation [134].

### 5.3. NK Cells

Natural killer (NK) cells are a subset of cytotoxic lymphocytes that differentiate from the common lymphoid progenitor cell and help compose the immune response to viral-infected and tumor cells. NK cells may be activated by cytokines, such as IL-2, IL-12, IL-15, and IL-18, or by the recognition of cells that are missing MHC class I surface molecules [87]. Activation triggers the release of cytotoxic granules that induce cell lysis or apoptosis [87]. Allogeneic MSCs have been shown to inhibit resting NK cell proliferation induced by IL-2 but had a limited effect on active NK cell proliferation [135]. This same study demonstrated that IL-2-activated NK cells efficiently lyse autologous and allogeneic MSCs, but this lysis was inhibited when MSCs were exposed to IFN-*γ*, presumably due to the upregulation of HLA class I molecules on MSCs [135]. Another study demonstrated an inverse correlation between HLA class I expression on MSCs and lysis by NK cells [136]. A more recent study demonstrated that priming with Toll-like receptors (TLR), specifically TLR3, may play a role in decreasing allogeneic MSC susceptibility to IL-2-activated NK cell killing [137].

### 5.4. B Cells

B cells differentiate from the common lymphoid progenitor cells and function in the humoral immunity of the adaptive immune response by the production of antibodies. Early studies demonstrated that murine allogeneic MSCs had inhibitory effects on the proliferation, activation, and IgG secretion of B cells [138]. Allogeneic MSCs inhibit B cell proliferation by inducing cell cycle arrest in the G0/G1 phase and by the production of soluble factors [139, 140]. Allogeneic MSCs were also shown to modify the activation pattern of the extracellular response kinase 1/2 and the p38 mitogen-activated protein kinase pathways, which are both involved in B-cell viability, activation, and proliferation [140]. Another study suggests that MSCs mediate their inhibitory effects on B cells through maturation protein-1 expression [141]. Allogeneic MSC inhibition of B cell activation seems to be dependent on IFN-*γ* and cell-to-cell contact via PD-1/PD-L1 interaction, in a similar fashion to MSC immunosuppression of T lymphocytes [142]. Contradictory data exists in which MSCs promote the proliferation and differentiation of B cells* in vitro* [143].

In summary, the immunosuppressive effects of allogeneic and autogeneic MSCs on immune cells are dependent on both the elaboration of soluble mediators as well as cell-to-cell contact. A high MSC to lymphocyte ratio also appears to be necessary to exert these effects, signifying a dose-dependent phenomenon.

The soluble mediators that function in the immunomodulatory role of MSCs with regard to the immune system have overlapping roles with the immunomodulation of bone cells, namely, osteoclasts and osteoblasts. Activated immune cells mediate increased bone turnover during inflammatory states; thus it seems plausible that the inhibitory effects of MSCs on these cells would promote an osteogenic state.

A key concern is that of negative regulation to prevent overimmunosuppression. In other words, great care should be taken to prevent complete suppression of the immune system, which could facilitate tumor formation or increased susceptibility to opportunistic infections.

It is likely a complex combination of synergism and antagonism among these various mechanisms that function to regulate the immune response. It is important to take into consideration that the aforementioned investigations were all conducted in* in vitro* settings, which may fail to include integral factors that are present in the* in vivo *milieu. Additionally, there are likely other unaccounted for factors that are specific to species, tissue, and experimental methods.

## 6. Role of Immune Cells and Cytokines in Modulating Osteogenic Differentiation of MSCs* In Vitro*

As discussed in Section 3, data from studies on human patients and experimental animals reveal that immune cells and cytokines produced by them, particularly T cells, IFN-*γ*, and TNF-*α* inhibit fracture healing and MSCs-induced bone formation. Treg cells and Th2 response seem to promote bone formation. However, the role of the Th2 response was reported in relation to ectopic bone formation and needs further validation in a fracture model. While IFN-*γ* and TNF-*α* induced apoptosis of MSCs* in vitro*, which can explain inhibition of bone formation by these cytokines as recently reported, molecular mechanisms of immune cell regulation of bone formation remain largely unknown. Since the immune response is typically mounted sequentially—first the attack of innate immune cells (macrophages, monocytes, and NK cells), followed by the adaptive immune response (antigen presenting cells, CD4^+^ T cells, CD8^+^ T cells, and B cells)—and early responding cell types can alter response by T and B cells, it is necessary to understand how each cell type interacts with MSCs* in vitro*.

A study by Omar et al. demonstrated that human monocytes stimulated by either LPS or IL-4 communicate proosteogenic signals to allogeneic MSCs, as evidenced by the increased expression of run-related transcription factor 2 (Runx2), alkaline phosphatase (ALP), and bone morphogenetic protein-2 (BMP-2) [144]. Since IL-4 stimulation primarily induces a Th2 response, this study suggests that the Th2 response would promote bone healing. Conditioned medium from cultures of human monocytes derived macrophages, however, was shown to suppress BMP-2-induced osteogenic differentiation of allogeneic MSCs, and this effect was associated with high levels of IL-1*β* and TNF-*α* [145]. Several studies indicate an osteogenic role for monocytes and macrophages alike [146–148]. For example, Oncostatin M, a member of the IL-6 family of cytokines, produced by activated macrophages was identified as a key player in inducing osteoblast differentiation from allogeneic MSCs while also inhibiting adipogenesis [146, 147]. A more recent study provides supporting evidence that monocytes and macrophages induce osteogenic differentiation and proliferation of human allogeneic MSCs via the production of BMP-2 [148]. Nicolaidou et al. demonstrated that monocytes and macrophages potently induced human allogeneic MSC differentiation into osteoblasts, mediated by cell contact, the production of monocyte soluble factors, and the activation of MSC STAT3 signaling by monocyte production of OSM [147]. Another study demonstrated that LPS-stimulated monocytes induced osteogenesis from human allogeneic MSCs via exosomes that resulted in the increased expression of Runx2 and BMP-2 [149].

T cell subsets are reported to differently regulate osteogenic differentiation of human MSCs* in vitro*. Conditioned medium from human CD4^+^ T cells but not from CD8^+^ T cells was shown to significantly upregulate the expression of Runx2, osteocalcin, ALP, and bone sialoprotein of allogeneic MSCs, as well as increase the mineralization in osteogenic cultures of MSCs [150]. MSCs were shown to phagocytose apoptotic cells and this phagocytosis enhanced osteogenic differentiation of MSCs [151]. Apoptotic cells treated MSCs expressed CXCR4 and CXCR5 which might enable them to migrate towards inflamed sites such as fracture repair or arthritic joints. These MSCs also secreted IL-8, MCP-1, and RANTES that can induce chemotaxis of T cells [151].

## 7. Conclusion

Immune cells and the cytokines that they produce play an important role in bone healing. Along with growth factors, the cytokines also guide differentiation of osteoprogenitor MSCs. Although inflammation plays a key role in fracture repair, particularly during the initial and remodeling phases of healing, chronic exposure to lymphocytes and to inflammatory signaling have been shown to impair the fracture repair process. The role of various immune cells and their subtypes in bone healing is complex and not completely understood. Therefore, thorough understanding of the immune cells control of fracture healing and precise ways to control the immune cells will be necessary when modulating the inflammatory response as potential new therapy for bone tissue engineering. MSCs can be effectively used for this purpose since they possess abilities to modulate immune cells differentiation and functions in specific microenvironments.

With this in mind, we propose the following areas as key topics of future investigations in the field: investigating interaction between MSCs and immune cells, particularly T cells and their subtypes* in vitro* and* in vivo* [150, 151], developing noninvasive techniques for imaging trafficking and activation of immune cells [152], and investigating local and systemic delivery of immune cells modulating agents (Treg cells [153], cytokine specific antagonists [152], corticosteroids [154], and nonsteroidal anti-inflammatory drugs [155]) to enhance bone healing and to study mechanistic aspects of correlation between inhibition of specific immune cells activities and bone healing.

## Figures and Tables

**Table 1:** List of mediators MSCs use to modulate proliferation and function of T cells.

Mediator	Target Cells	Modulation	Reference
Inducible nitric oxide (iNOS)	T cells	Inhibition of proliferation induced by anti-CD3 antibody	[28]

Programmed death ligand-1 (PD-L1)	T cells	Inhibition of proliferation induced by anti-CD3 antibody	[29]

Prostaglandin E2 (PGE2)	T cells	Inhibition of proliferation induced by anti-CD3 antibody	[30–33]

B7-H4 (Negative co-stimulatory molecule)	CTLs	Induces cell cycle arrest	[34, 35]

Fas ligand (Fas L)	T cells	Transient T cell apoptosis	[36]

TGF-*β*, human leukocyte antigen-G5 (HLA-G5), Notch1 ligands, heme oxygenase-1 (HO-1)	CD4^+^ T cells	Induction of Treg phenotype	[31, 37–41]

Chemokine receptor 6 (CCR6), and CD39	Th17 cells	Induction of Treg phenotype	[42–44]

ICAM-1, VCAM-1	T cells	Inhibition of proliferation through cell-cell contact	[45]

EP4 receptor, PD-L1, IL-10	T cells	Inhibition of Th17 differentiation	[32, 46, 47]

Galectins	T cells	Inhibition of proliferation	[48–50]

Indoleamine dioxygenase (IDO)	Human T cells	Inhibition of proliferation	[51–53]

MMP-2, MMP-9	Activated T cells	Cleavage of IL-2 receptor (CD25) on T cell surface leading to inhibition of proliferation	[54]
